# Bayesian Classification Models for Premature Ventricular Contraction Detection on ECG Traces

**DOI:** 10.1155/2018/2694768

**Published:** 2018-05-10

**Authors:** Manuel M. Casas, Roberto L. Avitia, Felix F. Gonzalez-Navarro, Jose A. Cardenas-Haro, Marco A. Reyna

**Affiliations:** ^1^Facultad de Ingenieria, Universidad Autonoma de Baja California, Mexicali, BC, Mexico; ^2^Instituto de Ingenieria, Universidad Autonoma de Baja California, Mexicali, BC, Mexico; ^3^Computer and Electrical Engineering and Computer Science Department, California State University, Bakersfield, CA, USA

## Abstract

According to the American Heart Association, in its latest commission about Ventricular Arrhythmias and Sudden Death 2006, the epidemiology of the ventricular arrhythmias ranges from a series of risk descriptors and clinical markers that go from ventricular premature complexes and nonsustained ventricular tachycardia to sudden cardiac death due to ventricular tachycardia in patients with or without clinical history. The premature ventricular complexes (PVCs) are known to be associated with malignant ventricular arrhythmias and sudden cardiac death (SCD) cases. Detecting this kind of arrhythmia has been crucial in clinical applications. The electrocardiogram (ECG) is a clinical test used to measure the heart electrical activity for inferences and diagnosis. Analyzing large ECG traces from several thousands of beats has brought the necessity to develop mathematical models that can automatically make assumptions about the heart condition. In this work, 80 different features from 108,653 ECG classified beats of the gold-standard MIT-BIH database were extracted in order to classify the Normal, PVC, and other kind of ECG beats. Three well-known Bayesian classification algorithms were trained and tested using these extracted features. Experimental results show that the F1 scores for each class were above 0.95, giving almost the perfect value for the PVC class. This gave us a promising path in the development of automated mechanisms for the detection of PVC complexes.

## 1. Introduction

According to the World Health Organization, cardiovascular diseases (CVD) are the main cause of death worldwide. An estimated 17.5 million people died from CVD in 2012, representing 31 of all global deaths [1]. The latest standard in the American Heart Association (AHA) on ventricular arrhythmias and sudden cardiac death in 2006, the epidemiology of ventricular arrhythmias includes a series of risk factors and clinical applications. These arrhythmias range from premature complexes, ventricular tachycardia and sustained ventricular tachycardia in individuals without cardiac issues background to sudden death due to ventricular tachyarrhythmia [2]. The electrocardiogram (ECG) is the main tool for the prediagnosis of heart diseases. Today, computer-aided analysis of short time ECG records, taken from supine positions, is a well-established procedure.

A normal heartbeat (NB) reflects a heart regular activity condition. On the other hand, premature ventricular contraction (PVC) is a kind of arrhythmia caused by an ectopic cardiac pacemaker located in the ventricle. PVC is a type of ECG arrhythmias that is identified for presenting anomalies in the normal cardiac rhythm, generating alterations in the heart rate that disrupts the mechanic and electric heart activity due to these delayed contractions (premature). On the ECG, these PVCs are characterized by premature and bizarrely shaped QRS complexes, usually wider than 120 ms, and a T wave larger than usual. A PVC event can be seen in healthy people and/or persons with some cardiac disorders, normally asymptomatic. The bizarrely shaped QRS complexes can increase the risk of a cardiac arrest and eventually may lead to a sudden cardiac death [3]. A bigger problem, however, is to track the presence and number of arrhythmias over days, weeks, and months. Since cardiologists cannot spend a lot of time in the analysis of millions of heartbeats from an individual, it is necessary to use automated mathematical algorithms to detect these abnormal events [4].

There have been so many improvements in ECG conditioning; some of these are signal-to-noise ratio enhancement, wave detection characteristics, heart rate variability analysis, and ECG patterns classification, among others. Since the new algorithms are increasingly more powerful and precise, gaps between the use of recent algorithms and the standard analysis methodology of the available evidence have begun to emerge [5–7]. Nazarahari et al. [8] used a neural network (NN) to classify six relevant types of heartbeats from a set of features created by new wavelet functions along with different distances and principal component analysis (PCA) to reduce the dimensionality. Martis et al. [9] applied the bispectrum computation in each beat and PCA to create the features that ultimately fed NN and support vector machines (SVMs) algorithms to classify between five types of heartbeats including the normal heartbeats (NBs) and premature ventricular contraction (PVC) beats. Afkhami et al. [10] derived morphological, statistical, and temporal features from the heartbeats amid probability density function extracted from the Gaussian mixture modeling (GMM) parameters to train an ensemble of decision trees. Javadi et al. [11] extracted features using the wavelet transform from key morphological shapes of the ECG and combined negative correlation learning with mixture of experts to train a negatively correlated NNs (neural networks). Kamath [12] used the Teager energy operator to derived nonlinear components in time and frequency; consequently, he fed a NN classifier to make predictions for five different arrhythmia beats. Martis et al. [13] segmented the QRS wave from each beat, derived features using the discrete wavelet transform (DWT), and compared PCA, linear discriminant analysis(LDA), and independent component analysis (ICA), three different dimensionality reduction techniques, to obtain the best method with greater performance classifying heartbeats. Sharma and Ray [14] put every heartbeat through the Hilber-Huang transform for feature extraction along with other set of features as statistical features, Kolmogorov complexity and weighted mean frequency which served as training for a SVM classifier. Banerjee and Mitra [15] proposed heuristic classification based on the cross wavelet transform of ECG signals to classify between abnormal and normal heartbeats. Oliveira et al. [16] designed a dynamic Bayesian network, and with a threshold set by an expert, it is able to classify between PVC and other kinds of beats.

These works give valuable information about the insights of the ECG nature and classification boundaries of the heartbeats and have a high classification performance. In this work, we compared three generative classifiers to distinguish between NB, PVC, and others. We attempted to simplify the feature extraction and use much more simple Bayesian generative model algorithms, Gaussian Naïve Bayes (GNB), Gaussian linear discriminant analysis (LDA), and quadratic discriminant analysis (QDA). In the case of the GNB, it assumes independence between features in such a way that every feature is parametrized by univariate *μ* and *σ*, and the LDA/QDA takes into account the joint distribution of the features, and these are parametrized by *μ* and ∑. These parameters are much more simple to understand, interpret, and correlate with the labels in question and are preferred over a complex hyperparameters as those can give much more detailed information about the characteristics, attributes, or components from the recollected and extracted data.

## 2. Materials and Methods

In this work, we considered the MIT-BIH arrhythmia database, available in the PhysioNet web page [17, 18]. It consists of 48 half-hour signal records of two channel ambulatory ECG recordings, digitized at 360 samples per second with 11-bit resolution over a 10 mV maximum range. The most important part of this database is that it has reference notations at each beat done by expert cardiologists.

**Figure 1:**
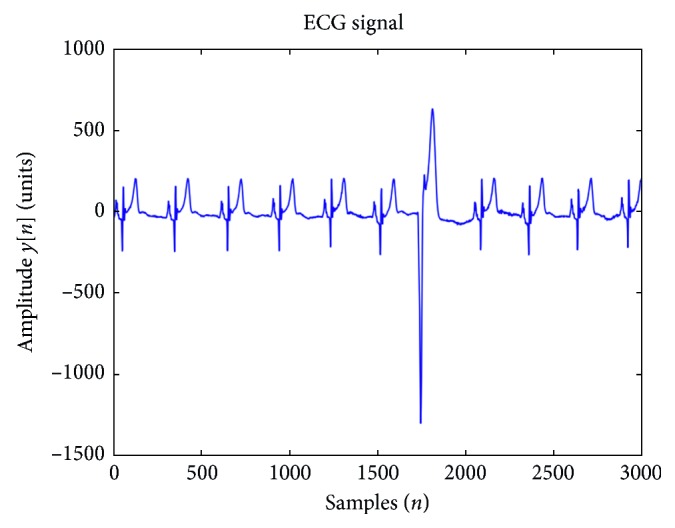
Samples of NB and an isolated PVC.

All the experiments were performed in MATLAB® 2014. For research purposes, along with the MIT-BIH arrhythmia database, the PhysioNet web page provides a file for every ECG record with a beat classification. We relabeled this database for this work in a similar way as in [8–16]. We kept the original labels of NB and PVC, and we mapped the rest of the heartbeats as “Others Beats” (OB). A total of 74,924 beats are classified as NB, 7129 beats as PVC, and 26,600 as OB. Giving a total of 108,653 beats for multiclass classification purposes. Samples of these beats can be seen in Figure 1.

**Figure 2:**
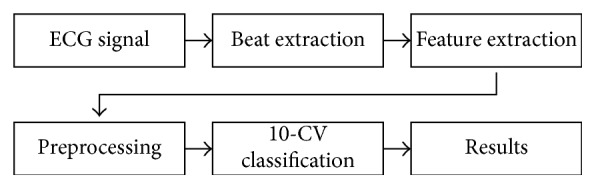
Workflow of the experimentation.

In Figure 2 is shown the workflow that we followed in this research. First, we extracted every beat from every signal in the database. Then, we put the data through a series of preprocessing steps that include the normalization, the transformed space, and the outlier detection. We proceeded with the experimentation using the generative models and the cross validation for the fair and safe comparison of our results.

### 2.1. Beat Extraction

For feature extraction purposes, we segmented each heartbeat taking a time window of 0.2 ms from R peak backwards and 0.46 from R peak forward, lasting 0.66 ms, which is approximately what a normal beat lasts. In this way, we created a matrix where every row was a heartbeat and the columns represented each sample point.

### 2.2. Preprocessing and Extraction of Features from ECG Beats

In order to train the classifiers and have a better performance, we processed the signals. The first step was to mean normalize every sample of every signal, and that was done with the following equation:

(1) $$\begin{array}{c} x_{\text{normalized}}=\frac{x_{\text{sample}}-x_{\mu }}{x_{\max}-x_{\min}}, \end{array}$$

where *x*_sample_ corresponds to the sample in a particular ECG signal, *x*_*μ*_ is the mean of signal, *x*_max_ is the maximum value of ECG signal, and *x*_min_ is the minimum value of the signal where the sample is located.

**Figure 3:**
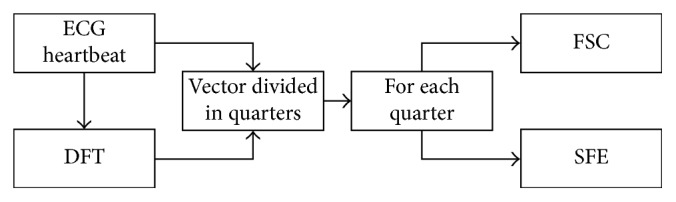
Feature extraction.

The main feature extraction is explained in Figure 3. Once the signals were mean normalized, each one was divided in four vectors. Each quartered vector was under a processing procedure for the extraction of 20 features. For each quartered vector, we used a procedure that we named “Feature Statistic Calculation” (FSC) where we calculate the mean, the standard deviation, and the maximum and minimum values. Also, for each quarter, we used another procedure that we named as “Samples Features Extraction” (SFE) where we extracted six samples that characterized the quartered vector. These six characteristic samples are the beginning and the ending of the quartered vector and four samples equally spaced from the quartered vector. The discrete Fourier transform (DFT) was applied in each of these quartered vectors, and for the resulting transformed quartered vectors, the same procedure of implementing the FSC and SFE was performed. At the end, as a result, we have extracted ten features from the quartered vector of the original heartbeat plus another ten features from the transformed quartered vector. As we divided all the signals in four vectors, we ended up with 80 features to feed the classifiers.

A great number of elements that has nothing to do with a disease can distort the ECG signals. The easier ones to remove with digital signal processing techniques (such as filters) are the 60 Hz powerline frequency and muscle-noise signals. However, as these data come from ECG Holters (a wearable device that records the signal from a patient through a considerable number of hours), many of heartbeats and the signal itself are disturbed by muscle movements, and these heartbeats recorded are very different in shape from the ones recorded before or after by standard methods. Using an outlier algorithm that helped our algorithm to be correctly trained as the models used are based on Gaussian distribution, outliers can affect greatly the performance of the model. The next step in the procedure was to get rid of the samples considered outliers. For this task, an algorithm which is based both in the multivariate Mahalanobis distance and in the comparison of the critical value of the *χ* ^2^ distribution was used [19]. For this, we needed to assume that the dataset behaves as a normal distribution; then, the Mahalanobis distances from every feature sample follow a Chi-square distribution with *d* grades of liberty, in this case 80. Any value above the 97.5th quantile is considered an outlier. The dataset was reduced to 84,586 beats, with 58,049 normal beats, 5,222 PVC beats, and 21,315 beats classified as others.

The “cluster-based visualization with scatter matrix” algorithm [20] was implemented in order to visualize and project the data in such way that it could be better classified. The scattering of matrices is a technique used to reduce the dimensionality and to maximize the dispersion between groups. However, this method is for clustered or labeled datasets. It is well known that the total variance *S*_t_ can be scattered in the sum of two terms called scattered matrices, which calculate the variance within the group *S*_w_ and the variances between each group *S*_b_. Due to the importance of the media within each cluster as representatives of each group, it is natural to project the information in a subspace covered by the media of each group. This can be done defining a set of orthonormal vectors ${\hat{b}}_{i}=1,\;\ldots ,\;N_{c}$ (*N*_c_ number of clusters) using the method of Gram–Schmidt orthogonalization:

(2) $$\begin{array}{c} X^{c}=\sum_{i=1}^{N_{c}}\left(X\cdot {\hat{b}}_{i}^{T}\right)\cdot {\hat{b}}_{i}, \end{array}$$

with *X* being the whole dataset. A way to preserve as much separation as possible between the groups, after the projection of the dataset into a subspace covered by the medias of each group, is through the process of whitening before this procedure:

(3) $$\begin{array}{c} X_{\text{esf}}=\alpha \cdot \beta^{-1/2}\cdot X. \end{array}$$

**Figure 4:**
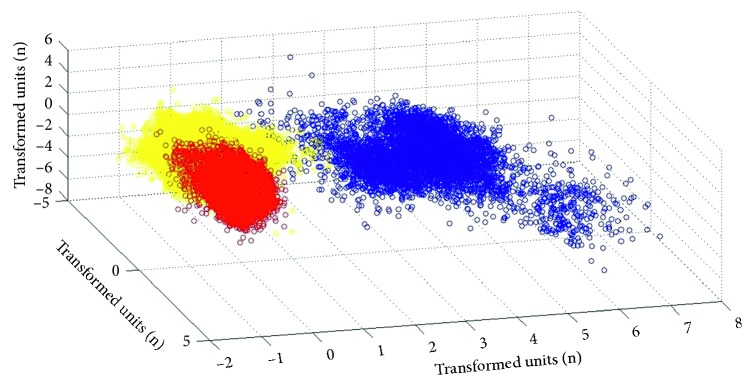
Clusters after classifications.

This last implementation is with the only purpose to reduce the dimensionality and visualization, and it does not intervene or affect in the process of clustering, where alpha and beta are the eigenvectors and eigenvalues, respectively. The computation of the scatter matrices for each class is by the following equations:

(4) $$\begin{array}{c} S_{w}^{C}=\sum_{j=1}^{N_{c}}\sum_{i=1}^{N_{j}}\left({\left(X_{i}^{C}-\mu_{j}^{C}\right)}^{T}\cdot \left(X_{i}^{C}-\mu_{j}^{C}\right)\right),\\ S_{B}^{C}=\sum_{j=1}^{N_{c}}\left(N_{j}\cdot {\left(\mu_{j}-\mu \right)}^{T}\cdot \left(\mu_{j}-\mu \right)\right),\\ M^{C}={\left(S_{w}^{C}\right)}^{-1}\cdot S_{B}^{C}, \end{array}$$

where *X*_*i*_ is the dataset of *i*th cluster, *μ* is the general mean, and *μ*_*i*_ is the mean of each cluster. Consequently, the diagonalization of the new scatter matrix *M* ^C^ shows that for each class, typically, the major part of the information is within the first eigenvalues. This eigenvector forms the second and third dimension for visualization purposes. The first three lower dimensional projections are shown in Figure 4; this image shows that there are some clear boundaries and three different types of beats, the normal, the PVC, and the others. The dataset was divided uniformly into 70, 15, and 15, for the training set, the validation set, and the testing set, respectively, without repetitions.

### 2.3. The Classifiers and the Classification

Once the data is prepared and ready to be used as a training set for the classifiers, we can make inferences with the Bayesian generative models. The Bayes theorem is defined as

(5) $$\begin{array}{c} P\left(q_{k}\left|X,\;\;\theta_{k}\right.\right)=\frac{p\left(X\left|q_{k},\;\;\theta_{k}\right.\right)\cdot \left(q_{k}\left|\theta_{k}\right.\right)}{p\left(X\right)}. \end{array}$$

For classification purposes, the divisor or the marginal probability is not necessary because it plays a constant role, and as we are interested to know which class *q*_*k*_ have a higher probability, then *P*(*q*_*k*_|*X*, *θ*_*k*_) is proportional to *p*(*X*|*q*_*k*_, *θ*_*k*_) · (*q*_*k*_) for every class:

(6) $$\begin{array}{c} P\left(q_{k}\left|X,\;\;\theta_{k}\right.\right)\propto p\left(X\left|q_{k},\;\;\theta_{k}\right.\right)\cdot \left(q_{k}\left|\theta_{k}\right.\right), \end{array}$$

where *P*(*q*_*k*_|*X*, *θ*_*k*_)  is the posterior probability, in this case, the class *q*_*k*_ given a sample *X* or a feature vector following a Gaussian distribution with parameters *θ*_*k*_; *p*(*X*|*q*_*k*_, *θ*_*k*_) is the  likelihood of the sample *X*, given a class *q*_*k*_ following a Gaussian distribution with parameters *θ*_*k*_; and *p*(*q*_*k*_)  is the probability that the class *q*_*k*_ is presented.

For numerical stability, we can use the logarithm operator, and now we can represent the classifier as a sum of logarithms:

(7) $$\begin{array}{c} \log\left(P\left(q_{k}\left|X,\;\;\theta_{k}\right.\right)\right)\propto \log\left(p\left(X\left|q_{k},\theta_{k}\right.\right)\right)+\log\left(p\left(q_{k}\left|\theta_{k}\right.\right)\right). \end{array}$$

The probability of the priori for each class is given by the ratio *P*=*n*_*c*_/*N*, where *n*_*c*_ is number of times *c* class is presented, and *N* is the total number of instances. This remains constant and only the likelihood changes for every classifier, as it modeled different.

As we have numerical data and in GNB assume that every feature is independent, the likelihood is formed by the multiplication of every feature parameterized by univariate Gaussian distribution's *μ* and *σ*. LDA and QDA are represented by *μ* and ∑, as these classifiers model the joint distribution of the features, and the likelihood term is modeled by the multivariate Gaussian distribution (MGD) with *d* dimensions and *k* classes and is defined by the following equation:

(8) $$\begin{array}{c} \mathrm{MGD}=-\frac{1}{{\left(2\pi \right)}^{d/2}{\left|\sum \right|}^{1/2}}e^{-\left(1/2\right){\left(x-\mu_{k}\right)}^{T}\Sigma^{-1}\left(x-\mu_{k}\right)}. \end{array}$$

And substituting it in likelihood in (7), we have

(9) $$\begin{array}{c} q_{k}\left(x\right)=-\frac{1}{2}\log\left|\sum_{k}\right|-\frac{1}{2}{\left(x-\mu_{k}\right)}^{T}\sum_{k}^{-1}\left(x-\mu_{k}\right)+\log\left(p\left(q_{k}\left|\theta_{k}\right.\right)\right), \end{array}$$

which leads to the QDA classifier.

If we assume the same covariance for all the classes, this enables us to use a linear classifier for each class:

(10) $$\begin{array}{c} q_{k}\left(x\right)=x^{T}\sum^{-1}\mu_{k}-\frac{1}{2}\mu_{K}^{T}\sum^{-1}\mu_{k}+\log\left(p\left(q_{k}\left|\theta_{k}\right.\right)\right). \end{array}$$

### 2.4. Evaluation of the ECG Classifier

Before explaining the experimentation process, the evaluation needs to be explained. The efficiency of a test is entirely captured by the following four basic measurements: true positive (TP), false negative (FN), false positive (FP), and true negative (TN). From these four basic measurements, all the other statistical measures can be derived. In this context, a true positive means that a PVC was predicted and the arrhythmia really happened, while a true negative means that a PVC was not diagnosed and the arrhythmia was, indeed, not present. However, a false positive means that a PVC was identified, but it really did not happen. Finally, a false negative is that a PVC was not detected although the arrhythmia really was there. Sensitivity (Se) or Recall indicates the ability of a test to identify positive cases; a test with high sensitivity has few false negatives. Positive predictive value (PPV) or Precision provides the probability of being true positive when the test is positive. Equations (11) and (12) show how to calculate the above-mentioned measurements:

(11) $$\begin{array}{c} \mathrm{Se}=\frac{\mathrm{TP}}{\mathrm{TP}+\mathrm{FN}}, \end{array}$$

(12) $$\begin{array}{c} \mathrm{PPV}=\frac{\mathrm{TP}}{\mathrm{TP}+\mathrm{FP}}. \end{array}$$

For the relation between these two parameters, we used the F1 score implemented as a good evaluator in [21] and can be expressed as

(13) $$\begin{array}{c} F=\frac{2\cdot \mathrm{Se}\cdot \mathrm{PPV}}{\mathrm{Se}+\mathrm{PPV}}. \end{array}$$

In the worst-case scenario, the F1 score is zero if the two parameters are zero; and in the best-case scenario, the F1 score is 1 if the two parameters are one. This ratio gives a good sense of how the algorithm does the classification.

### 2.5. Experimentation Set Up

The experiment was performed as follows: 10-crossfold validation performed for the classifier selection and assessment. The training and validation set were concatenated, and 10-crossfold validation was done for the three classifiers. We calculated the F1 score for every fold, and the mean was extracted in order to have an average of the performance from each classifier. Every class has a F1 score; in this way, every classifier has three F1 scores, and as a way to select the best among these, we calculated once again the mean from those results, and the model with the higher average was selected. With the training and validation set concatenated, the trained classifier chosen was tested then with the test set. The results from this experiment are shown in the following section.

## 3. Results and Discussion

Each record of the MIT-BIH Arrhythmia database was downloaded in the “mat” format. The extraction of every NB, PVC, and OB were just explained in the previous section. A dataset of 108,653 beats with 80 features were extracted, and after taking out the outlier samples, the database was reduced to 84,586 samples. For the training we used 70% of the data, for validation 15% of the data, and the remaining 15% was for testing purposes.

Three supervised learning algorithms were implemented to classify the ECG beats, and the results from this are shown in Table 1. It can be seen that the mean F1 scores for the Naïve Bayes is the lowest with a considerable 0.86. The top ones were the LDA and QDA, with mean F1 scores of 0.96 and 0.98, respectively. Obviously the QDA algorithm has the highest performance.

The new training set is composed of the training and validation sets, and it was used to train a new QDA model which was then tested with the remaining test set data. The results for this last algorithm are presented in Table 2. The performance of this last model is over 0.95 for sensitivity, recall, and F1 scores for each class. Also, the confusion matrix is added in the results Table 3; this shows which beats were classified correctly and which ones were not.

The present work proposes certain specific features as a way to generalize and reclassify the heartbeats. The results from the algorithms commonly applied give us an insight on the standard methodology followed in the classification of the heartbeats and in the degree of the complexity needed to discern between the classes even though there are some simple linear classifiers able to obtain high percentages in the classification rate.

However, there are much more complex models available with the capacity to learn even nonlinear features which are the state of the art in machine learning algorithms as is seen in Table 4. The classification difficulty varies depending on the kind of classes to detect; anyway most of them have already high percentages in their classification rates. The main goal of our work is not about the reaching of the 100 percent in the classification rate or to achieve better performance than the related works, but instead demonstrating that simple features can have unambiguous boundaries and that such features can reach a probability distribution which will then be helpful in giving insights about the different types of beats that we are trying to classify.

For example, in [8, 9, 12, 13], we use NNs to discriminate between the different types of beats according to their respective application. The multilayer NNs have been a great tool for classification purposes; they are powerful and their deep learning extension (state of the art for complicated problems like image classification and object detection on images) has put them into a powerful place to solve many problems. However, they are called “black box” algorithms since it is very hard to interpret the hyperparameters they learned through the training process. Also, the mixture of experts used in [11] with temporal-frequency domain features from the wavelet transform does not provide further insights of the classification boundaries which could be extrapolated into a medical interpretation. Although decision trees (applied in [10]) are known for being algorithms used in Business Applications for their great intuitive design and modeling, these along with the features extracted from HOS and GMM may represent a difficult task to find some interoperability; and Ensemble learning makes it even harder.

The SVM was considered state of the art for classification in its time, the kernel approach made suitable for tasks very hard to accomplish, and it is widely used nowdays as in [12, 14] but represents the same lack of interpretability for being a discriminant model along with the complicated feature extraction (Bispectrum, PCA transformations, and temporal-frequency coefficients of Hilbert-Huang transformation). Finally heuristic classification and dynamic threshold in [15, 16] works, respectively, depend on medical expertise to tune the decision. We believe that all these methods and approaches are suitable for the classification purpose, but we also believe that they overkill the issue. In our method, for all this, we get rid of all the unnecessary extra complexity, and we use simple Bayesian models adapted to explore and analyze the data using our proposed features for these classifications achieving very good results as shown.

The independence assumption, the linear and quadratic boundaries from the Naïve Bayes, the discriminant linear and quadratic classifiers, respectively, and the results from these classifiers give us an insight of the separation between classes with the features that we proposed. These algorithms make evident the advantage that there are no other hyperparameters to tune as in logistic regressions, in support vector machines, in neural networks, and so on. The results are showing us that the boundary among classes is not complicated for a linear classifier using the features proposed. The finding of promising algorithm candidates and methodologies to classify ventricular heartbeats, as well as normal heartbeats and other types of beats, can lead to better treatment and diagnosis of heart issues. Comparing our method with the results of other works puts our approach very close to similar implementations in which complex classifiers were used.

## 4. Conclusion

We evaluated the Naïve Bayes, LDA, and QDA algorithms to classify ECG beats in normal, PVC, and other kinds. These three models have high F1 scores, with Naïve Bayes presenting 0.86 while LDA and QDA presenting more than 0.95. However, in relation to the last two, the QDA classifier have a higher 0.983 F1 score. This performance results is the reason why we preferred the QDA classifier over the other three models. This chosen classifier was trained with both the training and validation sets, and it was tested with the corresponding data test set giving promising results. The F1 scores for each class were above the 0.95, giving almost the unit value for the PVC class; this was possible due to the fact that the relation of sensitivity and recall for this kind of beats yields better results than those in all the other classes. It can be proved from the confusion matrix that every PVC beat was correctly classified. The algorithms proposed in this work are, in nature, simple, and given that they are generative these assign a probability distribution to the features which can also give insights about the distinct heartbeats and their behavior. The finding of even more features for the mapping of these heartbeats into a better feature space and for their interpretation in behalf of the medical field is an active area of research.

## Figures and Tables

**Table 1:** Performance of each classifier tested.

Mean F1 scores for each train-validation test		
GNB	QDA	LDA
0.868	0.983	0.960

**Table 2:** Performance of the QDA classifier.

Performance of the final QDA model			
	Se	PPV	Fscore
NB	0.991	0.987	0.989
OB	0.959	0.974	0.967
PVC	1	0.980	0.990

**Table 3:** Confusion matrix of the classifier selected over the test set.

Confusion matrix of the final QDA model			
	NB	OB	PVC
NB	8698	78	1
OB	114	3014	14
PVC	0	0	769

**Table 4:** Comparisons between related works.

Comparison with other works							
Work	Year	Features	Classifier	Classes	Acc	Se	PPV
Nazarahari et al. [8]	2015	Wavelet + distances measures	Multilayer perception	Normal, PVC, APC, paced, LBBB, RBBB	97.51	—	—
Martis et al. [9]	2013	QRS, bispectrum, PCA	SVM NN	N, LBBB, RBBB, APC, VPC	93.48	—	—
Afkhami et al. [10]	2016	RR interval, HOS, GMM	Decision trees, ensemble learnes	AAMI, all classification in MIT-BIH	99.7	100	100
Javadi et al. [11]	2013	Wavelet + morpho-logical and temporal features	Mixture of experts, negative correlation learning	N, PVC, other	96.02	92.27	79.4
Kamath [12]	2011	Teager energy functions in time and frequency domains	Neural network	N, LBBB, RBBB, PVC, paced beats	100	100	100
Martis et al. [13]	2013	DWT + PCA + ICA + LDA	SVM, NN, PNN	AAMI	99.28	—	—
Sharma and Ray [14]	2016	Hilbert–Huang transform, statistical features	SVM	N, LBBB, RBBB, PVC, paced, APC	99.51	99.36	100
Banerjee and Mitra [15]	2014	Cross wavelet transform	Heuristic classification	Abnormal versus normal	97.6	97.3	98.8
Oliveira et al. [16]	2016	Dynamic Bayesian networks	Dynamic threshold	PVC versus others	99.88	99	99
Work		FSC, SFE	QDA	NB, PVC, OB	98.3	100	98
